# Association between genetically predicted rheumatoid arthritis and alopecia areata: a two-sample Mendelian randomization study

**DOI:** 10.3389/fimmu.2023.1269640

**Published:** 2023-10-31

**Authors:** Shengdong Zhong, Liting Lan, Zicheng Zheng, Huaiyuan Zhang, Yuqing Wen

**Affiliations:** ^1^ Department of Plastic Surgery, Longyan First Affiliated Hospital of Fujian Medical University, Longyan, China; ^2^ Clinical Research Center, The First Affiliated Hospital of Shantou University Medical College, Shantou, China; ^3^ Clinical Research Center, Longgang Maternity and Child Clinical Institute of Shantou University Medical College, Shenzhen, China; ^4^ Department of Orthopedics, Jinshan Hospital of Fudan University, Fudan University, Shanghai, China

**Keywords:** rheumatoid arthritis, alopecia areata, autoimmune disease, causation, Mendelian randomization, genetics

## Abstract

**Background:**

Although numerous observational studies have indicated a potential association between autoimmune diseases, such as rheumatoid arthritis (RA) and alopecia areata (AA), the research reports lack a clear causal relationship. In this study, our objective is to utilize the Mendelian randomization (MR) design to examine the potential causal association between RA and AA.

**Methods:**

To investigate the causal relationship between RA and AA, we utilized large-scale gene aggregation data from genome-wide association studies (GWAS), including RA (n=58,284) and AA (n=361,822) based on previous observational studies. In our analysis, we mainly employed the inverse variance-weighted (IVW) method of the random effects model, supplemented by the weighted median (WM) method and the MR Egger method.

**Results:**

The findings from the IVW methods revealed a significant association between genetically predicted RA and an increased likelihood of AA, as evidenced by an odds ratio of 1.21 (95%CI = 1.11-1.32; *P* < 0.001. Both the WM method and MR-Egger regression consistently showed significant directional outcomes (Both *P* < 0.05), indicating a robust association between RA and AA. Additionally, both the funnel plot and the MR-Egger intercepts provided evidence of the absence of directional pleiotropy, suggesting that the observed association is not influenced by other common genetic factors.

**Conclusions:**

The results of the study suggest a possible link between genetically predicted RA and AA. This finding highlights the importance for individuals diagnosed with RA to remain vigilant and aware of the potential development of AA. Regular monitoring and early detection can be crucial in managing and addressing this potential complication.

## 1 Introduction

Alopecia areata (AA) is a common cause of non-scarring hair loss and can present with a wide range of clinical manifestations, ranging from isolated patches of hair loss to complete loss of scalp hair (alopecia totals) or hair loss on the entire body (alopecia universalis). The incidence in the general population worldwide is estimated to be around 2% and has been increasing in recent years (1). Current available evidence suggests that AA can be considered as an autoimmune disease, which may result in significant psychological and emotional distress for affected individuals. Moreover, the impact of AA on patients’ lives is often underestimated.

Rheumatoid arthritis (RA) is an autoimmune disease, classified as one of the most prevalent chronic inflammatory conditions. Its primary impact lies within the joints, resulting in damage to cartilage and skeletal structures, as well as disability. Its incidence rate ranges from 0.5% to 1%. Considering its chronic nature, RA is linked to a decline in physical functioning and the emergence of complications, which consequently impose a substantial burden on both individuals and society (1). It is worth highlighting that recent years have witnessed a growing number of studies revealing a correlation between autoimmune disorders, such as RA, and hair loss (2). Epidemiological research demonstrates a significantly heightened risk of AA among individuals with autoimmune diseases, including RA (3). However, the causal relationship between RA and AA remains unclear to date, as observational studies are limited in their ability to establish causation due to uncontrolled and unmeasured confounding factors. To investigate this relationship, large-scale long-term randomized studies are required. However, conducting such studies presents challenges, as they are costly and maintaining long-term randomness is difficult. Hence, the primary objective of this study was to explore the association between RA and AA employing Mendelian randomization (MR) analysis (4). MR analysis is an innovative epidemiological approach that enables the evaluation of causal relationships between exposures and outcomes. A notable advantage of MR analysis is its ability to overcome the methodological limitations of observational studies, thereby diminishing the impact of potential confounding factors and reverse causality. This is due to the use of genetic variants as instrumental variables (IVs).

## 2 Materials and methods

### 2.1 Study design

**Figure 1:**
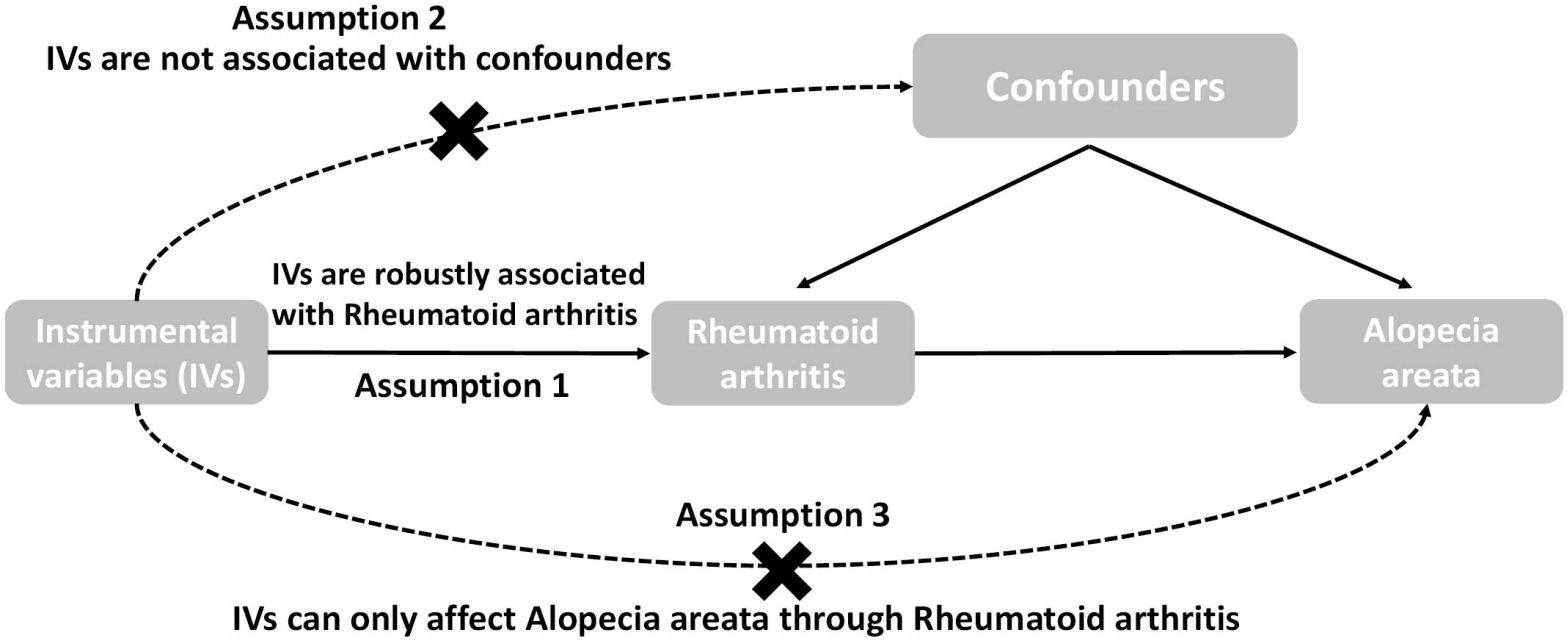
The overall design of the Mendelian randomization study: Two-sample MR studies need to satisfy three assumptions. Assumption 1: Instrumental variables should be associated with rheumatoid arthritis. Assumption 2: The selected instrumental variables should be independent of confounders. Assumption 3: Instrumental variables should affect outcomes only through exposure and not through direct correlation outcome merely through the risk factor, not via alternative pathways.

In this study, MR analysis utilized a collection of single nucleotide polymorphisms (SNPs) as IVs to estimate the causal effect of the exposure on the outcome. The key assumptions of MR analysis were identified as follows: firstly, the IVs should exhibit a significant association with the exposure being selected; secondly, the IVs must be independent of any potential confounding factors; and finally, the IVs should solely impact the outcome through their influence on the exposure, without any direct effect. These assumptions are crucial for the validity and reliability of MR analysis in determining causal relationships. The schematic representation of the study design is depicted in **Figure 1**. Two-sided p-values were utilized, with statistical significance set at *P* < 0.05. Statistical analyses were conducted using R software (version 4.2.2) and the TwoSampleMR package (version 0.5.0) (5).

### 2.2 Data sources for RA

The genetic associations of RA have been identified through a comprehensive genome-wide association study involving 58,284 individuals of European ancestry (https://gwas.mrcieu.ac.uk GWAS ID: ebi−a−GCST90013534). In a recent update conducted in 2020, Ha et al. expanded upon this research by investigating an additional 11 susceptibility sites of RA in both East Asian and European populations, utilizing a larger sample size in a genome-wide association study (6). In this study, a total of 14,361 cases and 43,923 controls from the European population were included in the analysis. The dataset comprised over 13 million imputed variants, which were carefully selected based on reliable imputation quality scores and a minor allele frequency (MAF) of ≥0.5%. To ensure accurate genetic information, whole-genome imputation was performed using the imputation reference panel derived from the 1000 Genomes Projects (1KGP) phase III data, with or without ancestry-matched whole-genome sequencing data. These meticulous steps were taken to enhance the accuracy and robustness of the study’s findings.

### 2.3 Data sources for AA

The genetic data for AA was obtained from the FinnGen biobank (https://www.finngen.fi/fi), a resource that has gathered biological samples from 500,000 participants in Finland over a duration of 6 years. On May 11, 2023, the Finn Gen Data DF9 was made accessible to the public, consisting of a vast cohort of 20,175,454 individuals and 2272 endpoints (7). To ensure accurate and reliable findings, we specifically utilized the data from the R9 release, which defines AA based on the International Classification of Diseases 8th to 10th codes. Prior to conducting our analysis, certain criteria were applied to refine the dataset. Individuals with ambiguous gender, high genotype missingness (>5%), excessive heterozygosity (4 standard deviations), and non-Finnish ancestry were excluded, resulting in a dataset comprising of 682 AA cases and 361,140 controls.

### 2.4 Instrumental variable selection

IVs estimation required for this study was obtained from the aforementioned Genome-Wide Association Studies (GWAS) results. To ensure the integrity of the IVs included in the MR analysis, we established the following screening criteria to extract eligible SNPs from the GWAS dataset, specifically focusing on RA. Firstly, we selected SNPs that exhibited a significant association with our exposure of interest (*P* < 5 × 10^-8^). Subsequently, we employed the European panel of the 1000 Genomes Project to prune the identified significant IVs within a 10,000kb window at a linkage disequilibrium (LD) r^2^ value of less than 0.001, in order to enhance the independence of the IVs. For each identified SNP instrument related to the exposure of interest (RA), we extracted the beta coefficient per allele, along with its corresponding standard error and p-value, from the AA GWAS dataset. IVs that exhibited a significant association with the outcome phenotype (AA) were excluded (*P* < 5 × 10^-8^). Subsequently, we harmonized the SNP instruments by aligning the directions of the alleles for both the Exposure-SNPs and the Outcome-SNPs, while eliminating incompatible or ambiguous SNPs with palindromic sequences (5). Finally, in order to minimize bias arising from weak IVs, an F statistic greater than 10 was deemed necessary. The calculation of the F statistics has been previously described (8).

### 2.5 MR analyses

In comparison to the conventional one-sample MR using two-stage least squares method, the two-sample approach generally exhibits enhanced statistical power and enables the utilization of extensive, unrelated datasets in order to initially obtain the instruments and subsequently examine the associations (9). Therefore, the study used a two-sample MR strategy to examine the relationship between SNP exposure and SNP outcome. The analysis employed three statistical approaches - inverse-variance weighted (IVW) (10), weighted median(WM) (11), and MR-Egger regression methods (12). These methods enabled the estimation of associations by utilizing summary statistics from distinct, independent samples. The IVW method involves the weighting of the Wald ratio for each SNP. The WM method provides a reliable estimate if at least 50% of the weight originates from valid (IVs. Additionally, MR-Egger regression allows for the exploration and adjustment of potential pleiotropic effects (5). The estimated effect size is presented as the odds ratio (OR; 95% confidence interval [CI]), which can be recorded per 1 standard deviation increase in RA with ORs of AA. All statistical tests were two-sided, and statistical significance was established at a threshold of *P* < 0.05.

### 2.6 Sensitivity analyses

Notably, the inference of causality in two-sample MR using multiple IVs could potentially be biased by pleiotropy. Hence, we performed sensitivity analyses to assess the dependability of our findings. Firstly, the MR-Egger method was utilized to investigate pleiotropy and determine if a single locus had an impact on multiple phenotypes. Pleiotropy refers to the occurrence of a single locus influencing multiple phenotypes. It was observed that horizontal pleiotropy undermines the validity of results obtained through MR analysis, highlighting the importance of considering and addressing this potential confounding factor in such studies (10). Horizontal pleiotropy is observed when the intercept term was significantly away from zero (intercept with *P* < 0.05) (13). Horizontal pleiotropy occurs when the intercept term significantly deviates from zero (intercept with *P* < 0.05) (13). Subsequently, we employed the MR-PRESSO approach to evaluate potential pleiotropy. This approach not only identifies and corrects possible outliers but also estimates causal effects (14). To assess heterogeneity in each MR association, we utilized Cochran’s Q statistic. The I^2^ statistic was employed to quantify heterogeneity between studies, where values below 25%, between 25% and 75%, and above 75% were considered indicators of low, moderate, and high heterogeneity, respectively (15). Visual assessment of heterogeneity was conducted using funnel plots. Furthermore, we conducted a leave-one-out analysis by systematically excluding each SNP and re-estimating the IVW estimation. Noteworthy changes in the results upon exclusion of a specific SNP suggest the presence of an outlier, calling for its elimination. The analyses were conducted using the “TwoSampleMR” package of R language (version 4.2.2).

### 2.7 Power calculation

Power calculation for the IVW estimates was based on a web tool at https://shiny.cnsgenomics.com/mRnd/ (16). Specifically, a sufficient statistical power is recommended to be over 80%.

### 2.8 Ethical approval

The data we used were obtained from published studies approved by the corresponding ethics committee, thus no further ethical approval was required for this study.

## 3 Results

### 3.1 The causal effect of RA on AA risk

A total of 77 independent SNPs were found to be significantly associated with RA based on the aforementioned screening criteria and no any SNP was found to be significantly associated with our outcome phenotype (AA) (**Supplementary Tables 1**). These 77 selected index SNPs explain about approximately 15.2% of the phenotypic variance of RA. Furthermore, the F statistics for these SNPs exceed 10, with values ranging from 30.0 to 1487.9 and an average of 116.4 (**Table 1**). This indicates that the genetic instruments used in this study possess substantial strength, essentially ruling out the potential for weak instrument bias (17).

**Table 1:** Association between rheumatoid arthritis and risk of alopecia areata using two-sample Mendelian randomization.

MR Methods	95%CL-	95%CL+	OR	beta	se	p
IVW	1.11	1.32	1.21	0.19	0.05	<0.001
weighted median	1.10	1.46	1.27	0.24	0.07	0.015
MR-Egger	1.05	1.36	1.19	0.18	0.07	0.011

**Figure 2:**
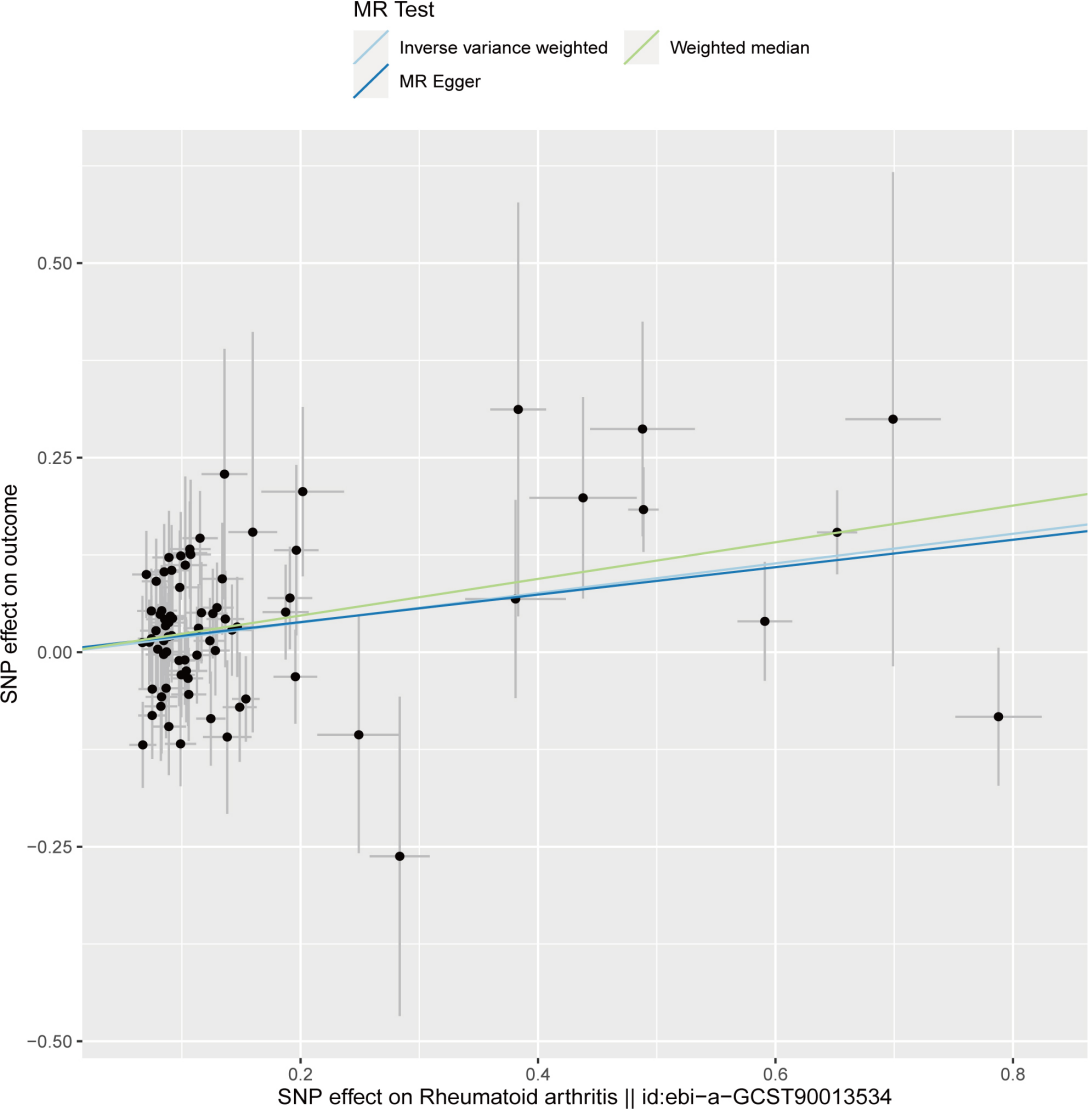
Scatter plot to visualize causal effect of rheumatoid arthritis on the risk of alopecia areata. The slope of the straight line indicates the magnitude of the causal association. The light blue line represents inverse-variance weighted (IVW), the light green line represents Weighted median, the dark blue line represents MR Egger.

**Figure 3:**
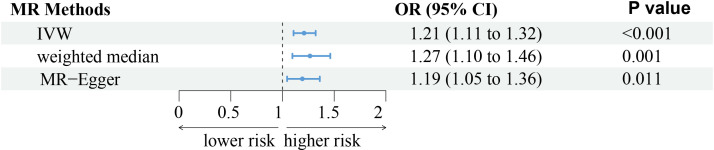
The forest plot represents the consistent results of this Mendelian randomization (MR) study in exploring the possibility of Rheumatoid arthritis to develop alopecia areata through three different methods. IVW, indicates inverse-variance weighted.

The analysis demonstrated consistent causal associations between RA and AA when employing various calculation methods (IVW, MR-Egger, WM). The slope of the scatter plot, illustrated in **Figure 2**, reflects the magnitude of the impact of RA on AA. These findings indicate that the risk of AA tends to increase with the severity of RA. Our preliminary analysis using the IVW method demonstrated a causal relationship between RA and an increased risk of AA (OR = 1.21, 95% CI = 1.11-1.32, *P <*0.001). Specifically, each standard deviation increase in RA was causally associated with a 21% higher risk of AA. However, it is worth noting that the power of the IVW estimate was observed to be low (57%). This may be attributed to the relatively small sample size of AA cases included in our analysis. Additionally, the MR-Egger and WM methods yielded consistent results (WM: OR = 1.27, 95% CI =1.10 -1.46, *P* = 0.001; MR-Egger: OR = 1.19, 95% CI = 1.05 - 1.36, *P* = 0.011) (**Table 1**; **Figures 2**, **3**).

### 3.2 Sensitivity analyses

**Figure 4:**
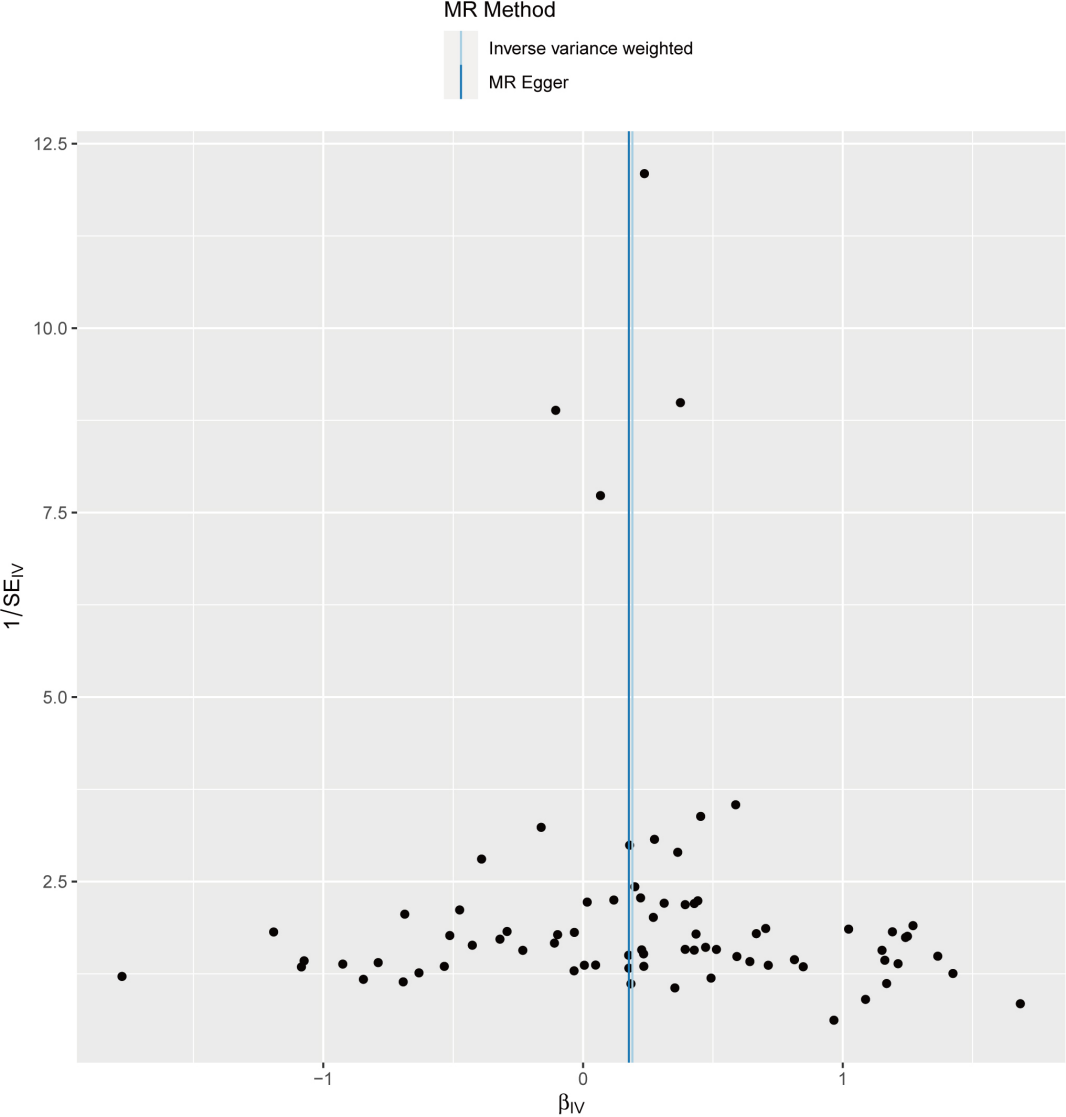
The funnel plot visually presents the overall heterogeneity of the Mendelian randomization (MR) estimates on the impact of rheumatoid arthritis on alopecia areata.

**Figure 5:**
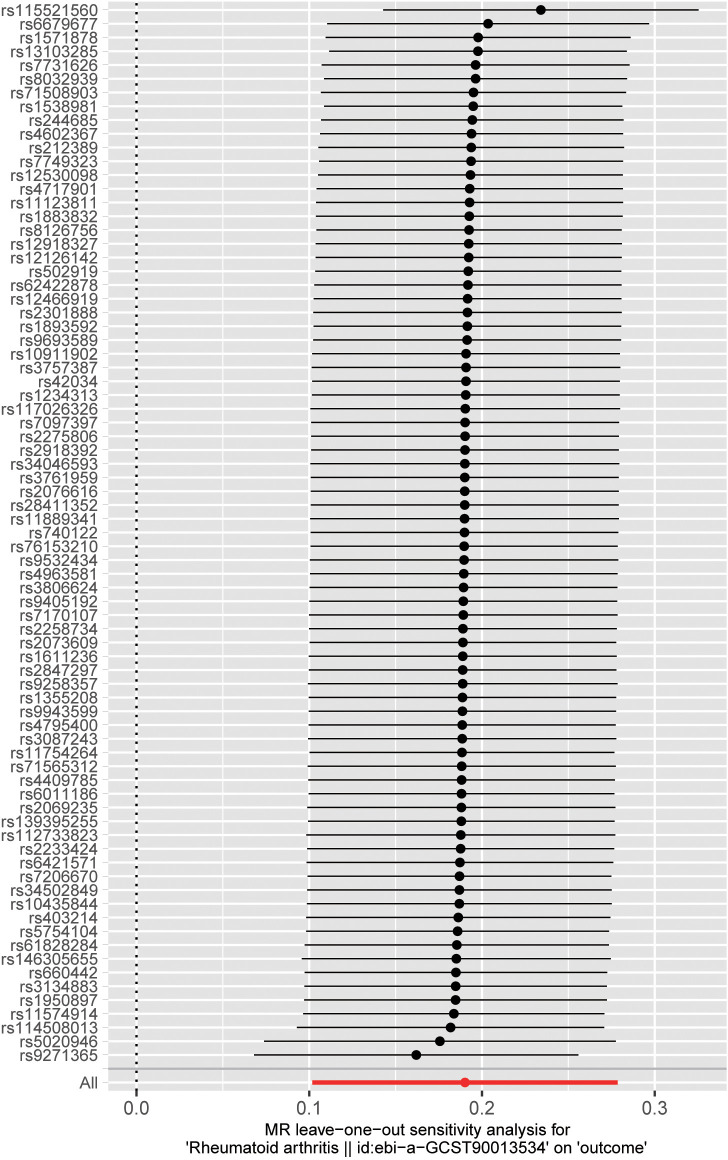
Visualize the impact of a single SNP on our research by creating a Leave-one-out plot.

We performed sensitivity analyses to check whether the association might have arisen through violations of the MR assumptions. No heterogeneity was observed in the Cochran’s Q-test (*P* > 0.05, I^2 ^= 19.39%), suggesting that the experimental findings are highly unlikely to be affected by heterogeneity. We found no evidence that our risk estimates were influenced by directional pleiotropy, as the average pleiotropic effect of the MR-Egger regression intercept was close to null (MR-Egger intercept: 0.003, *P* = 0.78) (**Figure 2**). In the MR-presso test, we also did not find the presence of outliers (abnormal SNPs) and horizontal pleiotropy. Visual assessment of directional pleiotropy using an MR funnel plot showed that variants were symmetrically distributed, indicating that our estimates were not driven by individual outliers (**Figure 4**). Finally, to investigate whether a single SNP had an outsized impact on the association, a leave-one-out sensitivity analysis was executed. The results, depicted in **Figure 5**, indicated that no individual SNP introduced bias into the association, supporting the robustness of the findings. Therefore, based on the present findings, we can reasonably infer that there is a substantial correlation between RA and AA and has a causal relationship.

## 4 Discussion

With a two-sample MR approach, our study demonstrated that patients with RA were causally associated with increased risks of AA, indicating that the autoimmune process of RA might help promote the occurrence and development of AA through immune surveillance. As far as we know, this study is the first MR study to examine the causal associations between genetic liability to RA and related traits with AA. The study design effectively minimizes the impact of potential confounders and inverse causation.

Numerous studies pertaining to autoimmune diseases have indicated that individuals with autoimmune diseases are more susceptible to developing AA. Even so, their association remains uncertain due to the conflicting outcomes and study design (18, 19). A retrospective study involving a sample size of 2613 individuals demonstrated that a history of autoimmune diseases increases the risk of all AA subtypes, including severe and localized forms (OR = 1.73; 95% CI = 1.10-2.72) (19).Although observational studies can establish correlations, due to reverse causal bias and the uncertain temporal order between AA onset and autoimmune diseases, it is currently insufficient to draw definitive conclusions about causal relationships in general research. In our MR study, we specifically analyzed the association between RA and AA among autoimmune diseases. The findings revealed a higher susceptibility of RA patients to develop AA, consistent with a prior cross-sectional study conducted in the United States (3). Similarly, a cohort study involving 2905 Asian RA patients also demonstrated a significant association between RA and AA. After adjusting for relevant factors, the risk of AA was 2.64 times higher (95% CI 1.47-4.76) in RA patients compared to those without RA (20).

It is noteworthy that in clinical practice, there is often little awareness of the direct correlation between RA and AA. Although the precise reason for this association remains unclear, we propose several hypotheses that may explain this trend. Firstly, both RA and AA are autoimmune diseases, and their pathogenesis may involve shared genetic and immune factors. Autoimmune diseases arise from abnormal attacks by the body’s immune system on its own tissues and organs. Consequently, patients with RA exhibit aberrant immune system activity, potentially rendering them more susceptible to developing AA. Secondly, inflammation plays a vital role in the pathogenesis of both RA and AA. RA is a chronic inflammatory disease, and AA is also associated with inflammatory reactions. Thus, the persistent inflammation in the bodies of RA patients may influence the development of AA. Finally, psychological factors may contribute to the susceptibility of RA patients to AA. The negative psychological stress, anxiety, and depression associated with chronic diseases may impact immune system function, thereby increasing the risk of developing AA. However, further research is necessary to validate these hypotheses. Understanding the precise reasons for this association is essential for improving clinical management strategies and enhancing patient prognosis. In summary, we opine that it is necessary and reasonable to enhance awareness and monitoring of AA development in RA patients during clinical practice.

There are several underlying mechanisms supporting explain the causality between RA and AA risks. The previous studies have demonstrated that Genetic markers in the TRAF1-C5 are associated with RA (21) and the polymorphism in this region increases the susceptibility and severity of RA (22). In a subsequent genetic study, it was found that TRAF1/C5 region may be involved in the etiology of AA, which indicates that there may be overlap of susceptible alleles between RA and AA, which provides some support for the study of the potential relationship between the two and the common etiology (23). In addition, an earlier mouse animal experiment showed that activation of the notch pathway can lead to additive differentiation of the adult hair shaft layers, leading to hair loss (24). In a Case–control study researchers found that the severity of AA was closely related to Notch4 gene (25). Then, in a case control analysis of healthy subjects including RA, AA and race matching, it was first proposed that there was a statistical association between RA and NOTCH4 polymorphism (26), This also provides certain evidence for the causal association between the two at the genetic level.

Furthermore, it is worth noting that GWAS have uncovered shared pathological changes in the Janus kinase(JAK) and signal transducer and activator of transcription signaling pathway in autoimmune diseases such as alopecia and RA (27). These changes can disrupt the regulation of immune responses, leading to immune dysregulation and the development and progression of autoimmune diseases. A clinical study involving 1307 RA patients demonstrated significant clinical improvement with the utilization of a JAK inhibitor (28). This improvement can be attributed to the crucial role of the JAK-STAT signaling pathway in the pathogenesis of RA, including synovial inflammation, autoantibody production, synovial proliferation, and joint destruction. Therefore, JAK inhibition emerges as a potential therapeutic approach to intervene and alleviate these pathological processes in RA. Considering the similar pathological alterations observed in both RA and AA, it is plausible to speculate that JAK inhibition may hold promise as a treatment option for AA as well (29). Furthermore, a study utilizing human clinical samples and mouse models has demonstrated that JAK signaling mediated by cytotoxic CD8 (+) NKG2D (+) T cells may contribute to the development of AA (30). Notably, three patients who received oral ruxolitinib (JAK1 and JAK2 inhibitors) treatment achieved nearly complete hair regrowth within 5 months, indicating the potential feasibility of JAK inhibitors in treating AA (31).

However, the direct correlation between cytotoxic CD8 (+) and RA is a subject of debate, despite the evident association observed between cytotoxic CD8 (+) and AA (30). Although recent studies have indicated a substantial increase in cytotoxic CD8 (+) levels among patients with RA (32), the precise connection between these two factors remains uncertain. Consequently, additional comprehensive investigations are imperative to unravel the shared immune regulatory cells that may exist between these two conditions in the future. In conclusion, these studies indicate that RA may play a positive role in the occurrence and development of AA, and our MR results can further confirm this and preliminarily explore its causal relationship. However, to validate the findings, it is crucial to conduct additional studies with a large sample size. Future studies should also incorporate the ability to assess nonlinear associations. Moreover, a comprehensive consideration of confounders, such as family history, personal lifestyle, and environmental factors, is essential. These endeavors will not only verify the results but also provide a deeper understanding of the underlying mechanisms involved.

This MR study has several strengths. Firstly, it is the first MR study to assess the causal relationship between RA and AA risks. The study’s main strength lies in its utilization of the MR design, which minimizes confounding and reverse causality. By leveraging the random allocation of genetic variants at conception, MR identifies causal effects that are less influenced by confounding factors and not prone to reverse causation. This approach enhances the study’s validity and strengthens the reliability of its conclusions (33). Secondly, this MR study utilized large-scale GWAS results from two independent populations of non-overlapping European ancestry, avoiding issues caused by population stratification and sample overlap. The results remained consistent across several sensitivity analyses, showing no evidence of heterogeneity or pleiotropy within our study (34). Additionally, the study focused exclusively on populations of European ancestry and employed genome-association tests that considered population structures. Consequently, the findings were not likely influenced by population stratification bias. However, it is important to note that these conclusions may not be generalizable to populations outside of European ancestry.

Several limitations of our study exist. In MR studies, the primary concern is horizontal pleiotropy, where genetic instrument variables affect the outcome via pathways unrelated to the exposure (35).And the MR-Egger intercept test does not indicate pleiotropy in our analysis, suggesting that there is no bias from pleiotropic effects in our study. These results support the conclusion that the observed outcomes are not influenced by alternative pathways and strengthen the validity of our findings. However, it is unsatisfactory that the power value calculated by our research institute is not entirely satisfactory (0.57 is less than 0.8), which may be due to the small sample size of GWAS data for AA. Therefore, in future studies, in order to obtain more accurate results, it is recommended to consider increasing the sample size to improve statistical power. In addition, the present MR analysis with summary-level genetic statistics was unable to estimate nonlinear association, and the assessment of gene-environmental interaction in summary-level data could not be performed. In summary, at the micro level, the intricate physiological mechanisms of RA and AA go far beyond these simplistic models. Further research is needed to identify potential mechanisms and gain a deeper understanding of AA for prevention. On a clinical level, larger sample sizes may be necessary for further investigations to validate our findings.

## 5 Conclusion

In conclusion, the results of this MR study indicate that RA is associated with an increased risk of developing AA. Based on these findings, we recommend that individuals diagnosed with RA exercise heightened vigilance for the possibility of AA and that screening recommendations be enhanced to enable early detection and prevention of AA in RA patients. However, it is essential to validate these findings through large-scale prospective studies and further investigate the underlying biological mechanisms involved.

## Data availability statement

The datasets presented in this study can be found in online repositories. The names of the repository/repositories and accession number(s) can be found in the article/**Supplementary Material**.

## Author contributions

SZ: Data curation, Visualization, Writing – original draft. LL: Data curation, Project administration, Visualization, Writing – review & editing. ZZ: Investigation, Writing – review & editing. HZ: Writing – review & editing. YW: Supervision, Writing – review & editing.
